# A case of diabetic ketoacidosis in a patient with COVID‐19 and newly diagnosed type 1 diabetes

**DOI:** 10.1002/ccr3.4881

**Published:** 2021-09-24

**Authors:** Keitaro Ishii, Hirotsugu Suwanai, Taishi Saito, Naoki Motohashi, Masaru Hirayama, Aya Kondo, Kouji Sano, Jumpei Shikuma, Rokuro Ito, Takashi Miwa, Ryo Suzuki

**Affiliations:** ^1^ Department of Diabetes, Metabolism and Endocrinology Tokyo Medical University Tokyo Japan; ^2^ Department of Emergency and Critical Care Medicine Tokyo Medical University Tokyo Japan

**Keywords:** continuous hemodiafiltration, COVID‐19, ketoacidosis, respiratory acidosis, SARS‐CoV‐2, type 1 diabetes

## Abstract

To improve severe ketoacidosis with COVID‐19, insulin treatment, invasive mechanical ventilation therapy, and continuous hemodiafiltration with sodium bicarbonate infusion were effective.

## Graphical abstract

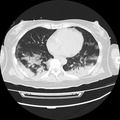

## 1 BACKGROUND

Coronavirus disease 2019 (COVID‐19) is a severe respiratory syndrome. The high ratio of diabetes was observed among patients with COVID‐19, but a few cases of type 1 diabetes (T1DM) with COVID‐19 have been reported. We experienced and rescued a case of newly diagnosed T1DM combined with COVID‐19. A 33‐year‐old woman was admitted to the hospital because of COVID‐19 and high glucose. She was diagnosed with T1DM according to laboratory findings. Among subtypes of T1DM, the clinical course was consistent with slowly progressive type 1 diabetes mellitus (SPIDDM), sometimes referred to as latent autoimmune diabetes in adults (LADA), rather than acute‐onset T1DM. Not only the metabolic acidosis attributable to ketoacidosis was observed in the patient, but also the arterial blood gas analysis (ABGA) was consistent with both a mixed respiratory and metabolic acidosis, together leading to severe ketoacidosis. Insulin treatment, invasive mechanical ventilation therapy, and continuous hemodiafiltration (CHDF) with sodium bicarbonate infusion were effective to improve severe ketoacidosis with COVID‐19.

The first case of coronavirus disease (COVID‐19) was reported in Wuhan, China, in December 2019. The World Health Organization (WHO) declared the COVID‐19 outbreak a global pandemic on March 11, 2020. COVID‐19 is caused by infection with a novel coronavirus, severe acute respiratory syndrome coronavirus 2 (SARS‐CoV‐2). COVID‐19 primarily manifests as upper respiratory symptoms; the symptoms may progress to pneumonia, which may lead to respiratory failure and, in severe cases, death. Several complications related to COVID‐19 have also been reported, including thrombotic events, central nervous system involvement, and cardiovascular complications.

By March 2021 in Japan, 436,055 cases of COVID‐19 have been diagnosed, resulting in 8297 deaths reported by the Ministry of Health, Labor, and Welfare. There are no data regarding how many of the total patients had diabetes mellitus (DM). In critical ill COVID‐19 patients, there is a high prevalence of DM. Indeed, DM is a poor prognostic factor for COVID‐19. Cases of type 2 diabetes mellitus (T2DM) with COVID‐19 have been reported, but few cases of newly diagnosed type 1 diabetes mellitus (T1DM) with COVID‐19 have been reported.^1^ Here, we discuss a case of newly diagnosed T1DM combined with severe ketoacidosis and COVID‐19.

## 2 CASE PRESENTATION

A 33‐year‐old woman was admitted to the hospital due to general weakness, polydipsia, polyuria, and dyspnea. Her eyes were closed, and she could not stand by herself and communicate appropriately. From her medical records, hyperglycemia was noted in a blood test about 2 years ago. She had a family history of T2DM, that is, her mother had T2DM, but did not have any family history of hypertension, dyslipidemia, or autoimmune diseases.

The diagnosis of COVID‐19 was confirmed at another hospital, 2 days before this hospital admission, based on real‐time reverse transcriptase‐polymerase chain reaction (RT‐PCR) assay for SARS‐CoV‐2. She was requested to stay at home until a scheduled admission was arranged. On the day of hospitalization, she was in shock with disordered consciousness and dyspnea.

## 3 INVESTIGATION

Her initial vital signs showed the following findings: blood pressure, low to unmeasurable levels; heart rate, 120 beats/min; respiratory rate, 24 breaths/min; and body temperature, 34.9°C. Her oxygen saturation was measured by pulse oximetry on a non‐rebreather (NRB) mask, with an oxygen flow rate of 10 L/min was 93%, and her Glasgow Coma Scale was 10 (E3V2M5). Upon admission, her plasma glucose level was 638 mg/dl and glycated hemoglobin level (HbA1c) was 15.7%, suggesting that she had untreated DM. Blood chemistry showed elevated D‐dimer levels at admission, which might be associated with the hypercoagulability of COVID‐19.

Arterial blood gas analysis (ABGA) on the NRB mask with an oxygen flow of 15 L/min showed acidosis with a pH of 6.74, pO_2_ of 79.3 mmHg, pCO_2_ of 37.7 mmHg, HCO_3_^−^ of 4.8 mmol/L, and anion gap of 27.2 mmol/L. The expected pCO_2_ was between 13.2 and 17.2 mmHg according to Winter's formula.^2^ However, the actual pCO_2_ was 37.7 mmHg, which was more than the expected pCO_2_, meaning that it was likely to represent combined metabolic and respiratory acidosis. In this case, we did not measure serum ketone levels.

**FIGURE 1:**
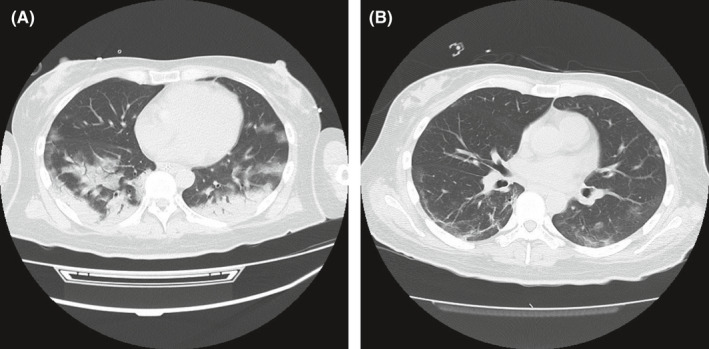
(A) Chest computed tomography on Day 1 of hospitalization showed bilateral distribution of GGO with consolidation in the posterior lobe and peripherally including crazy paving, air bronchogram, and a reticular pattern. (B) Improving bilateral GGO and fibrous stripes were observed in the lower lobes on Day 15. GGO, ground‐glass opacities

She was diagnosed with T1DM, as she had low insulin secretory capacity, ketoacidosis, and high titers of anti‐glutamic acid decarboxylase (GAD) and islet antigen 2 antibodies. Among the subtypes of T1DM, the clinical course was consistent with slowly progressive type 1 diabetes mellitus, sometimes referred to as latent autoimmune diabetes in adults, rather than acute‐onset T1DM^3^ (Table 1). Images of chest computed tomography on Day 1 of admission showed a crazy‐paving appearance, fibrous stripes, and characteristic of COVID‐19 pneumonia, and an air bronchogram suggested it was accompanied by bacterial pneumonia (Figure 1A). Blood, sputum, and urine cultures were negative for bacterial growth.

**TABLE 1:** Laboratory data

Urinalysis	Normal range	Admission (day 1)	Discharge(day25)
Protein (mg/dl)	Negative	100	N/A
Glucose (mg/dl)	Negative	2000	N/A
Ketone body	Negative	3+	N/A
Complete blood count			
White blood cell count (/µl)	2700–8800	24,900	4700
Red blood cell count (/µl)	3.7–5.4 × 10^6^	5.79 × 10^6^	3.90 × 10^6^
Platelet count (/µl)	140.0–340.0 × 10^3^	260 × 10^3^	401 × 10^3^
Hemoglobin (g/dl)	11.0–17.0	17.5	11.7
Hematocrit (%)	34.0–49.0	52.8	35.2
Biochemistry			
Aspartate aminotransferase (IU/L)	8–38	90	11
Alanine aminotransferase (IU/L)	4–44	25	5
Lactate dehydrogenase (IU/L)	106–211	1130	146
Amylase (U/L)	39–124	44	61
Lipase (U/L)	13–49	15.7	N/A
Urea nitrogen (mg/dl)	2.5–6.3	8.6	3.9
Sodium (mEq/L)	138–148	129	142
Potassium (mEq/L)	3.6–5.2	5.2	3.7
Chlorine (mEq/L)	98–108	97	108
Phosphate (mg/dl)	2.5–4.7	5.7	N/A
Blood urea nitrogen (mg/dl)	8.0–22.6	24.8	7.6
Creatinine (mg/dl)	0.4–0.8	0.63	0.38
D‐dimer (µg/L)	<0.80	28.14	0.69
Osmotic pressure (mOsm/L)	276–293	309	N/A
Infection			
Procalcitonin (ng/ml)	<0.5	4.47	N/A
C‐reactive protein (mg/dl)	<0.3	5.8	0.07
SARS‐CoV−2 RNA	Negative	Positive	Negative
Glucose Metabolism			
Glucose (mg/dl)	60–110	638	67
HbA1c (%)	4.6–6.2	15.7	N/A
Glycoalbumin (%)	11.8–16.3	46.7	N/A
C‐peptide (serum; ng/ml)	0.80–2.50	0.41	N/A
anti‐GAD antibody (U/ml)	<5.0	27.4	N/A
Anti‐insulin autoantibody (U/ml)	<0.4	<0.4	N/A
Islet antigen 2 antibody (U/ml)	<0.6	15	N/A
C‐peptide (urine; µg/day)	17–181	30.1*	N/A
Arterial blood gas analysis			
pH	7.35–7.45	6.74	N/A
pCO_2_ (mmHg)	36–44	36.7	N/A
pO_2_ (mmHg)	65–80	79.3	N/A
HCO_3_^−^ (mmol/L)	22–26	4.8	N/A
Base excess (mmol/L)	±2	−32.4	N/A
Lactate (mmol/L)	0.7–2.1	1.1	N/A
Anion gap (mmol/L)	10.0–14–0	27.2	N/A

Note

Laboratory data showed inflammatory reactions, hyperglycemia, thrombophilia, and severe acidosis on Day 1. ABGA was measured with the patient on a non‐rebreather mask with an oxygen flow rate of 15 L/min. The laboratory results were improved on Day 25. *Urine C‐peptide levels were measured on Day 18.

Abbreviations: ABGA, arterial blood gas analysis; GAD, glutamic acid decarboxylase; HbA1c, hemoglobin A1c; HDF, high‐power field; N/A, not applicable; PT‐INR, prothrombin time‐international normalized ratio.

## 4 TREATMENT

**FIGURE 2:**
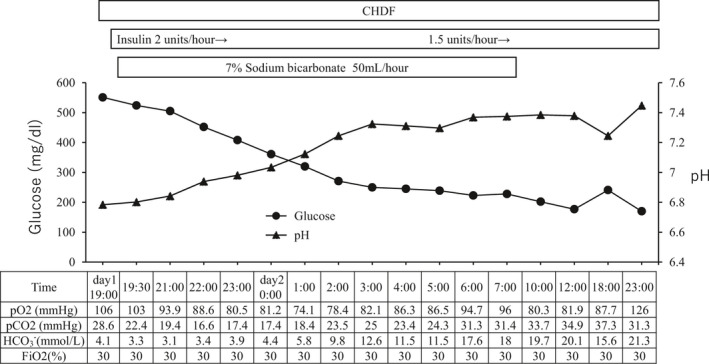
Time course of treatment at early stage of admission. Acidosis and ABGA were corrected with CHDF treatment, continuous intravenous insulin infusion, and sodium bicarbonate infusion. Glucose levels gradually decreased to the normal range. CHDF, continuous hemodiafiltration; ABGA, arterial blood gas analysis

She received continuous intravenous insulin infusion, invasive mechanical ventilation therapy, and continuous hemodiafiltration (CHDF) with sodium bicarbonate infusion to control her deteriorating respiratory condition and severe acidosis. The intravenous insulin infusion, starting from 2 units/h, was commenced immediately on admission until Day 14 (Figure 2). After obtaining written informed consent from her family, an antiviral agent, favipiravir, was administered for 14 days starting from Day 1 without adverse effects, such as liver failure and hyperuricemia. Ceftriaxone sodium hydrate, heparin sodium, and thrombomodulin alpha were administered on Day 1.

The patient's blood glucose levels decreased to the normal range within several days. Chest computed tomography showed improvement in the bilateral ground‐glass opacities (GGO) and fibrous stripes on Day 15 of hospitalization (Figure 1B). She resumed solid food eating on Day 16 and was treated with basal‐bolus insulin therapy. Her residual insulin secretion was reduced based on the low urinary excretion of C‐peptide on Day 18 (Table 1). The final total insulin dose was 30 units/day (0.48 units/kg), and each pre‐meal self‐monitoring of blood glucose levels ranged between 95 and 125 mg/dl. She gained weight in the range of 61.0–62.4 kg (her body mass index increased from 22.4 to 22.9 kg/m^2^) with continuous treatment of diet and insulin until day 26 of hospitalization.

## 5 OUTCOME AND FOLLOW‐UP

The RT‐PCR test results for SARS‐CoV‐2 were negative on Day 21 and Day 23 of admission, and she was discharged on Day 26. Blood examination at the follow‐up visit, 1 month after discharge, showed improved laboratory workup: serum C‐peptide (0.99 ng/ml), random plasma glucose (212 mg/dl), and HbA1c (6.9%). She continued to receive insulin therapy.

## 6 DISCUSSION

Patients with diabetes have higher susceptibility to, and severity of, COVID‐19 than non‐diabetes patients.^4^ Impairment of neutrophil function, such as defects in neutrophil chemotactic, phagocytic, and microbicidal activities, is considered as a possible etiology.^5^ Insulin therapy may be an effective way to achieve glycemic control and improve outcomes in patients with COVID‐19.^6^ Previous reports have shown that coagulopathy is common in patients with COVID‐19.^7^ In our case, antithrombotic therapy, heparin sodium, was administered on Day 1 of admission because her initial blood chemistry showed a high D‐dimer level of 28.14 μg/L, and the patient may have had thrombosis.

The acid‐base disturbance was a primary metabolic acidosis attributable to ketoacidosis. The initial ABGA was consistent with both mixed respiratory and metabolic acidosis.^2^

Upon admission, our patient's serum C‐peptide level was 0.41 ng/dl, which increased to 0.99 ng/ml 1 month after discharge, suggesting that her diabetes was in a transient insulin‐dependency state exacerbated by COVID‐19.^8^ Based on a multicenter surveillance study in the United States, 45.5% of COVID‐19–positive patients showed ketoacidosis with TIDM. Similar symptoms were observed, such as severe acute respiratory syndrome, including high blood glucose, elevated temperature, and dry cough.^9^, ^10^ High blood glucose levels accelerate symptoms of both COVID‐19 and T1DM, such as inflammation, hypercoagulability, hypoxia, fat catabolism, and ketoacidosis.^8^ In general, there are symptoms associated with virus and ketosis.

Inflammatory cascades, especially interleukin‐6, are involved in diabetic ketoacidosis (DKA). With regard to coronaviruses, it has been shown that SARS‐CoV‐1 binds to the angiotensin‐converting enzyme 2 receptor in the pancreatic islets and may damage them, causing acute‐onset diabetes.^9^ This leads to insulinopenia and an increased risk of DKA. Data on SARS‐CoV‐2 and DM are limited, and this mechanism remains speculative. In addition, patients taking sodium‐glucose co‐transporter‐2 (SGLT2) inhibitors are at risk of ketoacidosis; hence, they should discontinue this medication at the first sign of a severe flu‐like illness to prevent ketoacidosis.^9^

The course of TIDM caused by common enteroviruses (EV) often takes several weeks. In this case, although SPIDDM was in the background, T1DM worsened within a few days after infection, suggesting that the course of the disease was faster than that followed by EV infection. This may suggest that SARS‐CoV‐2 infects in cells of the human endocrine and exocrine pancreas.^11^

In conclusion, diabetes has been identified as a risk factor for a severe clinical course, coagulation, and ketoacidosis in COVID‐19 patients. However, there are many unexplained mechanisms; hence, more research is needed to better understand the underlying factors to control and prevent severe outcomes. The current case reports and a clear future direction about the immediate effects of SARS‐CoV‐2 on beta cells should be investigated.

## AUTHOR CONTRIBUTIONS

KI treated the patient, gathered information, and wrote the article. HS supervised and wrote the article. TS, NM, MH, AK, and KS treated the patient. JS, RI, and TM coordinated the medical treatment. RS supervised medical treatment. All authors contributed to the discussion and reviewed the manuscript.

## CONFLICT OF INTEREST

RS has received consulting and/or speaker fees from Novo Nordisk, Eli Lilly, and Sanofi.

## Data Availability

Data available on request from the authors.
